# Precision psychiatry requires disentangling disorder‐specific variation: The case of ASD

**DOI:** 10.1002/ctm2.1079

**Published:** 2022-10-10

**Authors:** Aidas Aglinskas, Stefano Anzellotti

**Affiliations:** ^1^ Department of Psychology and Neuroscience Boston College Boston Massachusetts USA

## 1 ENTANGLED VARIATION

Individual variation within autism spectrum disorder (ASD) is an established fact. Interventions that are effective for some individuals are not effective for others.^1^ Symptom severity, adaptive behavior scores and intelligence quotient partly correlate with differences in intervention outcomes, but a large amount of variation remains unexplained.^2^ Predicting how an individual would respond to different interventions is a key goal for clinical research. Such information could be used to recommend personalized care, curbing the process of trial and error and leading to benefits for the patients who would be able to access optimized interventions early. However, to make this possible, we need to gain a deeper understanding of individual variation within autism.

Neural data are a valuable source of information to make sense of individual variation. The brain mediates the relationship between genetics and behavior. Therefore, we can expect to observe individual variation within autism at the level of neural measures. Consistent with this, neuroimaging investigations have shown highly variable neuroanatomy among ASD participants.^3^ However, studying this variation has proven challenging: all brains (both with and without ASD) are unique and differ from another due to numerous genetic and environmental causes not related to ASD.^4^ Additional variation in common to ASD participants and controls is introduced by the process of measurement (for example, in large datasets different participants might be measured at different sites). *ASD‐specific* variation, therefore, is entangled with a large amount of variation from sources that are *shared* between typically developing individuals and those with ASD.

Traditional approaches, such as case‐control matching, can help to address these challenges when the factors that need to be matched are few and known. If some factors are unknown, however, it is impossible to match cases to controls along those factors. Furthermore, even if all factors are known, when there are many, it becomes extremely difficult to find controls that have the same specific *combination* of factors as a given case. Therefore, in order to study ASD‐specific variation controlling for shared variation, we need a different technique.

## 2 THE TECHNIQUE

Contrastive variational autoencoders (CVAEs^5^) are unsupervised deep learning models that take in samples from two populations, such as typical controls (TC) and ASD, and can be trained to isolate features that capture variation specific to one population (features that are ASD‐specific) from features that are common to both (features that are shared). Recently, we applied these models to a large database of neuroanatomical scans (ABIDE I,^6^ 512 ASD, 470 TD) extracting ASD‐specific features of neuroanatomy that vary within the ASD population.^7^ We show that the CVAE approach improves over previous methods of studying individual variation in ASD in several key areas.

**FIGURE 1:**
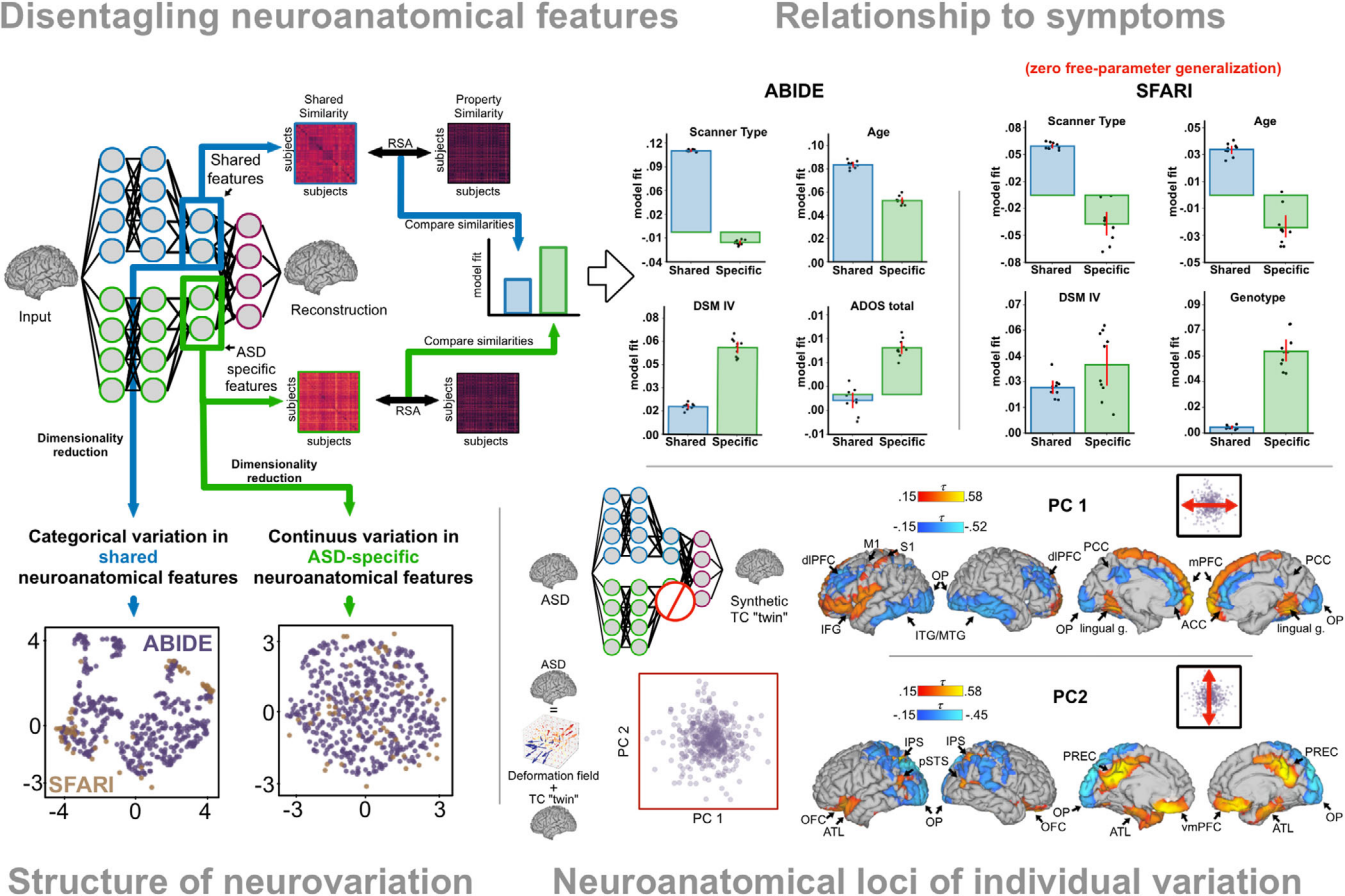
Disentanglement of neuroanatomical features using contrastive variational autoencoders (CVAEs). *Disentangling neuroanatomical features*: After training, CVAE separates neuroanatomical features into shared (blue outline) and ASD‐specific (green outline). *Relationship to symptoms*: Disentangled, ASD‐specific features correlate better with ASD‐related properties such as Diagnostic and Statistical Manual of Mental Disorders (DSM) IV behavioral subtypes, ADOS total scores and genotype associated with increased risk of ASD (16p11.2 deletion or duplication). Conversely, shared features correlate better with properties common to both ASD and typical controls (TC) participants (scanner type, age). *Structure of variation*: Shared features exhibit clustered structure, while ASD‐specific features exhibit continuous variation (confirmed using clustering analyses, not pictured). *Neuroanatomical loci of individual variation*: Reconstructing ASD brains using only shared features (synthetic ‘TC twin’) allows for precise neuroanatomical localization of individual variation in ASD. The first two principal components of this variation reveal a distributed set of regions that show expansion and contraction across individuals with ASD.

*Relationship between anatomy and symptoms*. Once disentangled from shared variation, ASD‐specific features of neuroanatomy correlate better with ASD symptom severity, such as Autism Diagnostic Observation Schedule (ADOS) scores (Figure 1). This is in contrast to models that do not disentangle (e.g., non‐contrastive VAEs) in which there is little to no relationship between neuroanatomy and symptoms. These findings underscore that relationships between neuroanatomy and ASD symptoms do exist but are easily overshadowed by ASD‐unrelated variation. Disentangling therefore seems to be necessary to reveal these relationships.

*Generalization*. CVAE derived, ASD‐specific features generalize to an independent dataset (SFARI VIP,^8^ Figure 1) without retraining the artificial neural networks. This is an important quality, because a model trained on one group of participants may need to be used to inform the diagnosis of new participants that were not included in the training dataset. The likely reason why ASD‐specific features learned by the CVAE generalize well is because the shared features capture common confounds affecting Magnetic Resonance (MRI) images, such as scanning‐site effects.

*Subtypes of autism*. Having established the validity of the ASD‐specific features and the reproducibility of the results, we used clustering analyses (Figure 1) to ask whether ASD‐specific features form neuroanatomical subtypes, as was previously theorized.^9^ Challenging previous findings, we found that, once disentangled from shared variation, neuroanatomical variation in ASD is distributed along continuous dimensions, rather than categorical subtypes (Figure 1). It is important to note that these results are specific to neuroanatomy: other data modalities might reveal the existence of clusters.

*Anatomical loci of variation*. The unique architecture of CVAEs enables precise neuroanatomical localisation of ASD‐specific variation. Like all variational autoencoders, CVAEs are generative models: given a set of features, they can reconstruct a brain image with those features. The brain image of an ASD participant, for example, can be reconstructed using a combination of its shared and its ASD‐specific neuroanatomical features. Repeating this process while setting the ASD‐specific features to zero allows for generating artificial brain images that are closely matched on shared features but lack ASD‐specific neuroanatomical features (‘synthetic TC‐twin’). This is a data‐driven evolution of traditional case‐control designs powered by deep learning that improves upon traditional case‐control designs (the latter cannot control for factors that are not explicitly matched). This comparison, between the ASD participants’ brains and their synthetic twins, revealed a broadly distributed pattern of neuroanatomical variation within ASD (Figure 1).

*Future outlook*. Disentangling ASD‐specific variation from shared variation overcomes a major hurdle in the study of individual differences within autism. However, a long road still separates us from the ambitious goal we set out at the beginning of this article—personalized care. First, extending the investigation of ASD‐specific variation to other data modalities, such as functional imaging and electroencephalography, will provide a more complete picture of individual differences within autism. Second, while we have found relationships between individual differences in neuroanatomy and symptoms, a key future step will be to establish relationships between variation in neuroanatomy and response to interventions. This will require the collection of new, large datasets that include neural measurements as well as intervention outcomes for hundreds of people. Finally, for some individuals, the most effective interventions might not yet have been invented. For this reason, a central challenge will be to rapidly incorporate newly developed interventions within this paradigm of personalized care, building infrastructure to integrate clinical practice with data collection and analysis.

## CONFLICT OF INTEREST

The authors declare no conflict of interest.
